# Clinical Updates and Perspectives on Transcranial Magnetic Stimulation (TMS)

**DOI:** 10.3390/jcm13133794

**Published:** 2024-06-28

**Authors:** Mariagiovanna Cantone

**Affiliations:** Neurology Unit, Policlinico University Hospital “G. Rodolico-San Marco”, 95123 Catania, Italy; m.cantone@policlinico.unict.it

Since its introduction nearly 30 years ago, Transcranial Magnetic Stimulation (TMS) has increasingly been used to both provide novel insights into the pathophysiology of the neural circuitry that underlies neurological and psychiatric diseases and to manipulate neural activities in a non-invasive manner [1]. Using dedicated TMS protocols, it is possible to explore the development of connections during brain maturation or that of connectivity during motor or sensory learning [2]. TMS is a neurophysiological looking-glass, enabling the detection of the reorganization of brain circuits in pathological conditions such as stroke [3], depression [4], and neurodegenerative brain diseases [5]. More recently, new and intriguing data have revealed its capability to identify subclinical patterns in sleep disorders [6] and systemic disorders [7]. Researchers are keen to discover the potential that lies in combining TMS with other functional brain investigations, such as electroencephalography or brain imaging. However, many aspects how TMS works are still being debated. This Special Issue will provide a collection of reviews and research articles about current and future trends in TMS.

The repetitive TMS (rTMS) paradigm, in which recurring magnetic impulses are delivered in rapid succession to the same cortical target, allows for the transient modulation of the stimulated cortical area’s functioning [8] through the induction/release of neurotrophins and angiogenic factors, changes in electrocortical excitability, and variation in cerebral blood flow, thus inducing short- and long-term neuroplastic phenomena [9,10,11,12].

In recent years, rTMS has been extensively studied to assess its potential to modulate cognitive function in mild cognitive impairment (MCI). MCI is a prodromal stage of dementia, characterized by subjective cognitive deficits and objective memory impairment without impairment in daily activity, since memory deficits are the clinical hallmark and the central characteristic of MCI. A recent systematic review published by Sharbafshaaer [13] confirmed that rTMS with low-/high-frequency stimulation in the left/right or bilateral dorsolateral prefrontal cortex (DLPFC) might have a positive effect on cognition (i.e., executive, memory, language, and visuospatial functions) and behavior abnormalities (i.e., apathy) in MCI patients, including those with vascular subtypes.

Moreover, TMS sheds light on the in vivo neurochemical mechanisms underlying cortical plasticity in patients with major depression. The rTMS protocol, with its wide range of applications in terms of therapeutic and rehabilitative purposes, received approval by the Food and Drug Administration in 2008 as an add-on therapy for adult drug-resistant MD [14]. In light of this, Almheiri and coworkers [15] retrospectively explored responses to rTMS treatment in a cohort of 505 patients with depression, aiming at directly comparing the response of different age groups with depression to rTMS and antidepressant treatment. The majority of the patients were treated with high-frequency protocols to the left dorsolateral prefrontal cortex (DLPFC). They observed that patients aged below or above 60 years did not differ significantly with respect to treatment efficacy, as indicated by absolute and relative changes to their HDRS-21 sum scores. Response and remission rates were around 30%. Elderly patients showed a significantly superior reduction in the item “appetite” and a superior reduction tending towards significance in the item “work and interests”. Their results support the use of rTMS in older adults utilizing the same protocols as in younger patients.

Interestingly, Ikawa and colleagues [16] used the TMS clinic registry database to compare clinical data from fifty patients with major depressive disorder (MDD) not treated with antidepressants and receiving TMS therapy as first-line treatment with data from fifty patients with MDD treated with the usual antidepressants, matched as closely as possible for age, gender, and depression severity. All patients included in the study received 20–30 sessions of intermittent theta burst stimulation (iTBS) therapy to the left DLPFC. The Montgomery–Åsberg Depression Rating Scale (MADRS) was used as the primary outcome in this registry data analysis study. There was no significant between-group difference in response rate, remission rate, or relative change in MADRS total score following TMS therapy, suggesting that iTBS monotherapy may be as effective as iTBS add-on therapy to antidepressants for patients with MDD, even when patients initially receive TMS therapy without taking antidepressants due to concerns about side effects from pharmacotherapy. Research aimed at disentangling the mechanism by which neuroplasticity plays a role in the pathological processes that lead to depression, as well as at evaluating the effects of neuroplasticity modulation, is needed for the field to facilitate more powerful translational research studies and identify novel therapeutic targets.

Moreover, the brief report by Roth [17] is a phase 4 study evaluating the safety and efficacy of deep TMS treatment in a large, multicenter cohort of late-life MDD patients. Deep TMS utilizes specially designed H-coils to stimulate the deep and broad cerebral regions associated with the reward system. The resulting improved depth of penetration may be particularly important in late-life MDD patients, who often experience brain atrophy. It is a well-designed study and a significant milestone in late-life MDD treatment research, demonstrating high response and remission rates in late-life MDD patients that promise a deeper understanding of treating this condition.

Finally, the study by Cosentino and coworkers [18] investigates the effects of rTMS administered in healthy subjects in the context of understanding the mechanisms of pain relief for patients with chronic pain conditions. It evaluates the offset analgesia (OA) phenomenon in healthy subjects. The thenar eminence was selected as the site of testing and the main outcome metric was VAS during heat application. The intervention consisted of applying rTMS to the primary motor cortex (M1) at 90% of the motor threshold. The findings were that, in this healthy patient population and under this study design, OA was not present; however, there appeared to be an emergence of OA with active rTMS, but not with sham treatment. The authors conclude that rTMS at M1 might have antinociceptive effects and call for further study in the chronic pain population.

Although rTMS has been approved or cleared for “safety and efficacy” in various therapeutic indications, there is still a need for substantially larger, rigorously designed studies [19]. Together with other non-invasive brain stimulation techniques [20] and conventional treatments, rTMS can be considered a reliable and translational strategy in patients with different neuropsychiatric disorders.
